# Neutralization of IL‐17A Ameliorates PM2.5‐Induced Cardiac Inflammation and Fibrosis in Rats by Suppressing the TXNIP/NLRP3 Signaling Pathway

**DOI:** 10.1002/kjm2.70316

**Published:** 2026-11-06

**Authors:** An‐Shuai Ba, Meng Wu, Wei‐Chen Li, Wei‐Yi Li, Di Ma, Yu Wang, Zhen‐Zhen Chen

**Affiliations:** ^1^ The First Hospital of Anhui University of Science and Technology Huainan China; ^2^ Anhui University of Science and Technology Medicine School Huainan China; ^3^ Mingguang People's Hospital Chuzhou China

**Keywords:** cardiac fibrosis, IL‐17A, inflammation, PM2.5, TXNIP/NLRP3

## Abstract

Fine particulate matter (PM2.5) pollution is a major environmental risk factor for cardiovascular diseases. Exposure can cause cardiac inflammation and fibrosis. However, whether interleukin‐17A (IL‐17A) contributes to these effects is unclear. Here, a rat cardiac injury model was established by intratracheal instillation of PM2.5, and an IL‐17A neutralizing antibody was administered. PM2.5 exposure increased the proportion of splenic CD4^+^IL‐17A^+^ T cells and IL‐17A/TRAF6 expression in cardiac tissue, with impaired cardiac function, myocardial inflammatory infiltration, collagen deposition, and increased expression of α‐SMA and Collagen I/III. Cardiac tissue exhibited increased oxidative stress, enhanced NF‐κB activation, elevated IL‐6 and IL‐1β, upregulated TXNIP, and activation of the NLRP3 inflammasome and pyroptosis‐related proteins (ASC, cleaved Caspase‐1 p20, GSDMD‐N, and IL‐18). Neutralizing IL‐17A reversed these abnormalities and improved cardiac function. In vitro, IL‐17A upregulated TXNIP/NLRP3 and increased the expression of inflammatory cytokines and extracellular matrix components in neonatal rat cardiac fibroblasts (NRCFs), whereas TXNIP knockdown reversed these effects. These results identify IL‐17A as a key driver of PM2.5‐induced cardiac inflammation and fibrosis, and targeting the IL‐17A–TXNIP/NLRP3 axis may offer a new approach to treating PM2.5‐related cardiac injury.

## 1 Introduction

Fine particulate matter (PM2.5) pollution is a significant environmental risk factor for cardiovascular diseases [1, 2]. PM2.5 can travel through the respiratory tract and reach the alveoli. It induces local pulmonary inflammation and can also enter the circulation. Thus, it can directly or indirectly damage the cardiovascular system [3]. Globally, PM2.5 exposure is associated with approximately 1.33 million deaths from ischemic heart disease and 32.17 million disability‐adjusted life years (DALYs). It is also associated with about 1.14 million deaths from stroke and 28.74 million DALYs [4]. Animal experiments have further demonstrated that chronic PM2.5 inhalation can induce myocardial interstitial fibrosis and promote ventricular remodeling [5]. Cardiac fibrosis is characterized by aberrant fibroblast activation and excessive extracellular matrix secretion. These changes increase collagen deposition in the myocardial interstitium. The resulting fibrosis impairs cardiac systolic and diastolic function [6].

Inflammation and excessive production of reactive oxygen species (ROS) are the main mechanisms underlying PM2.5‐induced cardiac fibrosis [7]. Toxic components concentrated on the surface of PM2.5 can activate pulmonary macrophages and epithelial cells. These cells release large amounts of pro‐inflammatory cytokines into the circulation. These cytokines then trigger local cardiac inflammation [8]. A persistent inflammatory microenvironment directly stimulates fibroblast activation. It also indirectly promotes fibrosis through pathways such as oxidative stress and pyroptosis [9]. Among inflammation‐related molecules, interleukin‐17A (IL‐17A) has been implicated in fibrosis across multiple organs [10, 11, 12]. IL‐17A is primarily produced by Th17 cells, but can also be secreted by γδT cells, innate lymphoid cells, and other cells [13]. In the diseased heart, IL‐17A participates in cardiac remodeling by promoting neutrophil recruitment, inducing fibroblast proliferation, and upregulating pro‐fibrotic factors [14]. Studies have reported increased IL‐17A expression in cardiovascular disease models including atherosclerosis [15], myocardial infarction [16], and hypertension [10]. Blocking IL‐17A signaling alleviates disease severity in these models. However, whether IL‐17A neutralizing antibodies confer protection against PM2.5‐induced cardiac inflammation and fibrosis remains unclear.

The NOD‐like receptor family pyrin domain‐containing protein 3 (NLRP3) inflammasome is a central effector in the innate immune system. Upon activation, it cleaves caspase‐1 and promotes the maturation and release of IL‐1β and IL‐18. The resulting cytokine release triggers sterile inflammation [17]. Thioredoxin‐interacting protein (TXNIP) is a key endogenous activator of the NLRP3 inflammasome. Under basal conditions, TXNIP binds to thioredoxin (Trx) and remains inactive. When intracellular ROS levels rise, Trx undergoes oxidation. TXNIP is consequently released. TXNIP then binds to NLRP3 and induces inflammasome assembly [18]. Studies have shown that PM2.5 exposure activates the cardiac NLRP3 inflammasome. Inhibiting this pathway reduces myocardial inflammation and fibrosis [7, 19]. However, the upstream signals that regulate the TXNIP/NLRP3 pathway in PM2.5‐induced cardiac injury remain unclear. Specifically, the involvement of IL‐17A in this pathway is unknown.

In this study, we induced cardiac injury in rats by intratracheal instillation of PM2.5. The intervention involved administration of an IL‐17A neutralizing antibody. We assessed histopathological changes, collagen deposition, cardiac function, oxidative stress markers, NF‐κB activation, and key proteins in the TXNIP/NLRP3 pathway. We aimed to investigate whether IL‐17A neutralization plays a protective role in PM2.5‐induced cardiac inflammation and fibrosis. In addition, we assessed the direct effects of IL‐17A on neonatal rat cardiac fibroblasts (NRCFs) in vitro. We examined cellular signaling changes following IL‐17A stimulation. These experiments helped clarify its cellular mechanism.

## 2 Methods

### 2.1 Preparation of PM2.5

PM2.5 samples were collected from Huainan, China, from December 2024 to May 2025. A high‐volume air sampler (Wuhan Tianhong Instruments, China) collected PM2.5 on glass fiber filters. The filters were cut into small pieces (1 × 1 cm^2^) and immersed in ultrapure water. The immersed filter pieces underwent three sonication cycles of 30 min each. The eluate was filtered through eight layers of gauze. The filtrate was freeze‐dried to obtain PM2.5 particles. The chemical composition of the collected PM2.5 particles was analyzed. The results are presented in Supplementary Figure 1. The dried PM2.5 was weighed and suspended in 0.9% normal saline. The mixture was sonicated for 30 min to produce a PM2.5 suspension of the desired concentration. The suspension was sterilized by exposure to an ultraviolet lamp for 30 min and stored at −20°C. For animal experiments, the PM2.5 suspension was thawed, diluted to the required concentration in a centrifuge tube, and sonicated again before use.

### 2.2 Establishment of the Rat Model

Six‐week‐old specific pathogen‐free (SPF) Sprague–Dawley (SD) rats were purchased from Beijing Vital River Laboratory Animal Technology Co. Ltd. (Beijing, China). Rats were maintained in an SPF facility with unrestricted access to food and water. Following a 2‐week acclimatization period (at 8 weeks of age), experiments began. Using the method reported by Zang et al. [20], myocardial fibrosis was induced through intratracheal instillation of PM2.5 suspension (10 mg/kg) under anesthesia with 5% chloral hydrate. Twenty‐four rats were randomly assigned to four groups (*n* = 6 per group): (1) Anti‐IL‐17A group: rats were given PM2.5 suspension by intratracheal instillation and a subcutaneous injection of an IL‐17A neutralizing antibody (1 mg/kg, Bio X Cell, USA, #BE0173); (2) IgG group: rats were given PM2.5 suspension by intratracheal instillation and a subcutaneous injection of an isotype control antibody (1 mg/kg, Bio X Cell, USA, #BE0088); (3) PM2.5 group: rats were given PM2.5 suspension by intratracheal instillation and a subcutaneous injection of an equal volume of sterile PBS; (4) Control group: rats were given an equal volume of sterile normal saline by intratracheal instillation and a subcutaneous injection of an equal volume of sterile PBS. Both intratracheal instillation and subcutaneous injection were administered twice weekly for 6 consecutive weeks (12 times in total). Within 24 h after the final intervention, echocardiography was first performed to assess cardiac function. Body weight was then recorded. The rats were then deeply anesthetized with an intraperitoneal injection of pentobarbital (150 mg/kg) and euthanized. The hearts were collected. The left ventricles were isolated, weighed, and stored at −80°C for further analysis.

### 2.3 Pathological Analysis

Heart tissue samples were fixed in 10% neutral buffered formalin at 4°C for 48 h. The fixed tissues were dehydrated through a graded ethanol series, cleared in xylene, and embedded in paraffin. Serial sections (approximately 4 μm thick) were prepared from these blocks. The sections were deparaffinized with xylene, rehydrated through a graded ethanol series, and then stained. Hematoxylin and eosin (H&E) staining (Boster, China) was used to evaluate general histopathological changes. Masson's trichrome staining (Boster, China) was used to assess collagen deposition. The collagen‐positive area in Masson's trichrome‐stained sections was quantified in ImageJ software as a measure of myocardial interstitial fibrosis.

### 2.4 Cardiac Function Assessment

Cardiac function was assessed with a Vevo 2100 ultrasound imaging system (FUJIFILM Visual Sonics, Canada). The measured parameters were left ventricular ejection fraction (LVEF), left ventricular fractional shortening (LVFS), left ventricular end‐diastolic diameter (LVEDD), and left ventricular end‐systolic diameter (LVESD). Additionally, we calculated the ratio of left ventricular weight to body weight.

### 2.5 Biochemical Analysis

Blood samples were collected from the retro‐orbital venous plexus via retro‐orbital puncture into coagulation‐promoting tubes. The samples were centrifuged at 4000 rpm for 10 min. Serum was obtained from each group. Biochemical parameters were measured with commercial assay kits for aspartate aminotransferase (AST; Fosun Diagnostics, China) and lactate dehydrogenase (LDH; Leadman, China).

### 2.6 Intracellular Cytokine Staining of Splenic Lymphocytes

Rat spleens were handled under sterile conditions, minced, and gently homogenized with PBS. The resulting homogenate was passed through a 70 μm cell strainer. Cells were pelleted by centrifugation at 1500 × *g* for 5 min at 4°C. The supernatant was removed. The pellet was resuspended in 2 mL of red blood cell lysis buffer (Absin, China) to lyse erythrocytes. Cells in the resulting single‐cell suspension were counted with a hemocytometer and the density was adjusted to 1 × 10^6^ cells/mL for subsequent experiments. Stimulation of splenic lymphocytes was performed in culture medium containing a protein transport inhibitor cocktail (BD Pharmingen, USA) for 4 h at 37°C in a 5% CO_2_ incubator. After stimulation, cells were collected by centrifugation at 1200 × *g* for 5 min at 4°C. The cell pellet was resuspended in 100 μL of PBS and stained with APC‐conjugated anti‐rat CD4 antibody (Thermo Fisher Scientific, USA) for 30 min at 4°C in the dark for cell surface staining. After PBS washing, cells were prepared for intracellular IL‐17A staining using a commercial fixation/permeabilization kit (BD Pharmingen, USA) following the manufacturer's instructions. Fixed and permeabilized cells were incubated with PE‐conjugated anti‐IL‐17A antibody (Thermo Fisher Scientific, USA) for 30 min at 4°C in the dark. Following staining, cells were resuspended in 1 mL of flow cytometry staining buffer. Fluorescence was recorded using a BD FACSCanto flow cytometer and analyzed using FlowJo software.

### 2.7 Elisa

Commercial ELISA kits (Boster, China) were used to quantify IL‐6 and IL‐1β in the supernatant of rat heart tissue homogenates. All assays followed the manufacturer's protocols. Absorbance at 450 nm was read using a microplate reader. Cytokine concentrations were calculated from standard curves generated with reference standards.

### 2.8 Oxidative Stress Assessment

Heart tissues were homogenized in normal saline and centrifuged at 2500–3000 rpm for 10 min. Supernatant levels of superoxide dismutase (SOD), malondialdehyde (MDA), and glutathione (GSH) were determined with the respective assay kits (Beyotime, China) according to the manufacturers' protocols. Total ROS in the homogenates was also assessed with a 2′,7′‐dichlorodihydrofluorescein diacetate (DCFH‐DA) fluorescent probe assay (Beyotime, China). Fluorescence intensity was measured at Ex/Em = 488/525 nm.

### 2.9 Immunohistochemical (IHC) Analysis

Paraffin‐embedded sections were deparaffinized, rehydrated, and subjected to antigen retrieval. Sections were incubated overnight at 4°C with primary antibodies targeting IL‐17A (Boster, China), TRAF6 (CST, USA), α‐SMA (CST, USA), Collagen I (CST, USA), Collagen III (Boster, China), and TXNIP (CST, USA). The sections were subsequently incubated with HRP‐conjugated secondary antibodies for 45 min at room temperature. After DAB color development, the percentage of the area with positive staining was calculated using ImageJ software.

### 2.10 Quantitative Real‐Time PCR (RT‐qPCR)

Total RNA was extracted from rat heart tissues or NRCFs using Trizol reagent (Sangon Biotech, China). First‐strand cDNA was produced by reverse transcription with the MightyScript Plus First‐Strand cDNA Synthesis Kit (Sangon Biotech, China) at 42°C. Quantitative real‐time PCR (qPCR) was performed with SGExcel FastSYBR Master Mix (Sangon Biotech, China) to quantify target gene expression. Amplification conditions for qPCR followed the manufacturer's instructions. *GAPDH* served as the internal reference gene. Relative target gene expression levels were calculated using the 2^−ΔΔCt^ method. Primer sequences are provided in Table 1.

**TABLE 1:** Primer sequences for quantitative real‐time PCR.

Gene	Forward primer (5′‐3′)	Reverse primer (5′‐3′)
*α‐SMA*	GAGGGATCCTGACCCTGAAG	CCACGCGAAGCTCGTTATAG
*Collagen I*	CCCTGCTGGAGAAGAAGGAA	AGGAGAACCTTTGGGACCAG
*Collagen III*	ACTGGTGAACGTGGCTCTAA	GGACCTGGATGTCCACTTGA
*TXNIP*	GAACCCACTCGGCTCAATCA	CGGCTTTGACTCGGGTAACT
*NLRP3*	ATTACCCGCCCGAGAAAGG	TCGCAGCAAAGATCCACACAG
*GAPDH*	GACATGCCGCCTGGAGAAAC	AGCCCAGGATGCCCTTTAGT

### 2.11 Cell Culture and Transfection

Primary NRCFs were isolated from SD rat pup hearts using established protocols [21]. Tissues were digested with 0.8 mg/mL trypsin and 0.5 mg/mL Type II collagenase, and the resulting cells were subjected to differential adhesion on uncoated dishes for 90 min, cultured in DMEM/10% FBS, and passaged twice. The passaged cells were then seeded. To assess the effects of IL‐17A, cells were treated with PBS (Control) or recombinant rat IL‐17A (100 ng/mL, Invitrogen) for 24 h. Separately, to examine the involvement of TXNIP, cells were transfected with siNC or siTXNIP (Shanghai GenePharma, China) using Lipofectamine RNAiMAX according to the manufacturer's protocol; after 48 h, the medium was replaced and cells in both transfected groups were exposed to IL‐17A (100 ng/mL) for another 24 h. Cells were harvested for subsequent analysis.

### 2.12 Western Blot

Total protein from rat heart tissues or NRCFs was extracted in RIPA lysis buffer containing 1% protease inhibitor and phosphatase inhibitor. Protein concentrations were measured with a BCA protein assay kit (Beyotime, China) following the manufacturer's instructions. Equal protein amounts were resolved by SDS‐PAGE and transferred onto PVDF membranes. Membranes were blocked in 10% non‐fat milk for 1.5 h at room temperature. Overnight incubation at 4°C was then performed with primary antibodies to IL‐17A (Boster, 1:2000), TRAF6 (CST, 1:1000), α‐SMA (CST, 1:1000), Collagen I (CST, 1:1000), Collagen III (Boster, 1:2000), p‐NF‐κB (Savier, 1:600), NF‐κB (Boster, 1:2000), IL‐6 (Boster, 1:2000), IL‐1β (Boster, 1:2000), Nrf‐2 (Boster, 1:2000), SOD1 (Boster, 1:2000), TXNIP (CST, 1:1000), NLRP3 (Boster, 1:2000), ASC (Proteintech, 1:10,000), Caspase‐1 (CST, 1:1000), GSDMD (CST, 1:1000), IL‐18 (Proteintech, 1:10,000), GAPDH (Boster, 1:10,000), and β‐Actin (Boster, 1:10,000). Following five washes in TBST (5 min each), membranes were incubated with the corresponding HRP‐conjugated secondary antibodies for 2 h at room temperature. An enhanced chemiluminescence (ECL) substrate was used to visualize protein bands for imaging with a ChemiDoc imaging system. The intensity of each band was quantified in ImageJ software.

### 2.13 Statistical Analysis

Data are presented as the mean ± standard deviation (SD). Data were analyzed with GraphPad Prism 10.1.2 software. Two groups were compared using an unpaired Student's *t*‐test. Multiple groups were compared by one‐way or two‐way analysis of variance (ANOVA), followed by Tukey's post hoc test. A *p* value < 0.05 indicated statistical significance.

## 3 Results

### 3.1 Increased Expression of IL‐17A and TRAF6 in the PM2.5‐Exposed Rat Model

TNF receptor‐associated factor 6 (TRAF6) is a key downstream component of the IL‐17A signaling pathway. Its K63‐linked ubiquitination and interaction with NF‐κB activator 1 (ACT1) mediate IL‐17A‐induced NF‐κB activation [22]. To assess the effects of PM2.5 exposure on IL‐17A and TRAF6 expression, we determined the proportion of splenic CD4^+^IL‐17A^+^ T cells by flow cytometry. We also measured protein levels of IL‐17A and TRAF6 in heart tissues by IHC and Western blot. The proportion of splenic CD4^+^IL‐17A^+^ T cells increased after PM2.5 exposure. This increase indicates a systemic IL‐17A response to PM2.5 (Figure 1A,B). IHC and Western blot analyses showed that protein levels of IL‐17A and TRAF6 in the heart were significantly higher in the PM2.5 group than in the control group. Both protein levels decreased markedly following administration of the IL‐17A neutralizing antibody (Figure 1C–H).

**FIGURE 1:**
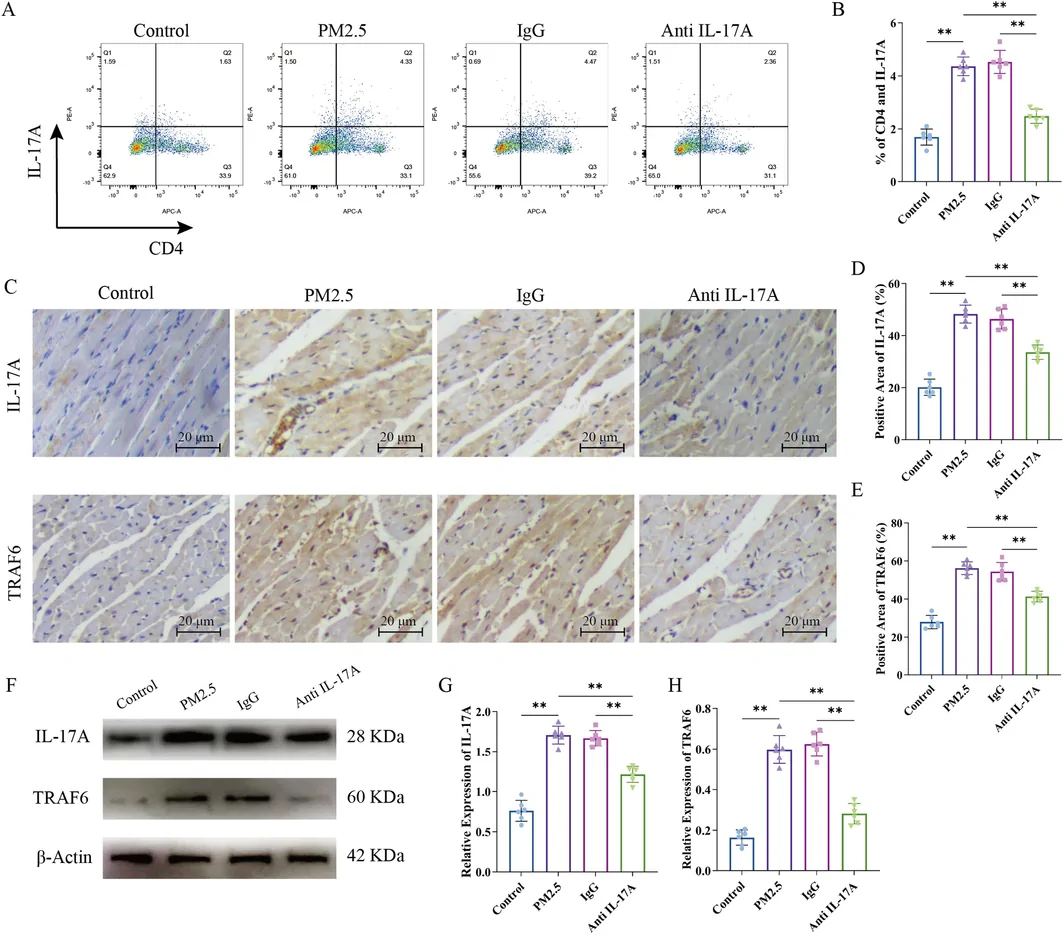
Expression of IL‐17A and the downstream signaling molecule TRAF6 in cardiac tissue. (A, B) Flow cytometric analysis of splenic lymphocytes; (C–E) IHC images and quantification of the positive area for cardiac IL‐17A and TRAF6; (F–H) Western blot analysis of cardiac IL‐17A and TRAF6. Data are presented as the mean ± SD (*n* = 6). **p < 0.01.

### 3.2 IL‐17A Neutralization Ameliorates PM2.5‐Induced Cardiac Pathological Changes in Rats

Previous studies have shown that high‐concentration PM2.5 exposure induces cardiac fibrosis in rats [20]. To assess the effect of the IL‐17A neutralizing antibody on PM2.5‐induced cardiac pathological injury (Figure 2A), heart tissues were stained with H&E and Masson's trichrome. H&E staining (Figure 2B) showed that in the Control group, myocardial fibers had a regular arrangement. The cytoplasm appeared abundant and homogeneous. The interstitium had a normal structure. In contrast, the PM2.5 and IgG groups exhibited marked myocardial disorganization and inflammatory cell infiltration. These injuries were significantly attenuated after treatment with the IL‐17A neutralizing antibody. The cardiac pathological injury score increased after PM2.5 exposure. It decreased following IL‐17A neutralizing antibody treatment (Figure 2C). Masson's trichrome staining (Figure 2B) revealed only sparse collagen fibers in the Control group. The PM2.5 and IgG groups showed increased blue collagen deposition and a larger fibrotic area. Treatment with the IL‐17A neutralizing antibody reduced collagen deposition and significantly decreased the fibrotic area (Figure 2D).

**FIGURE 2:**
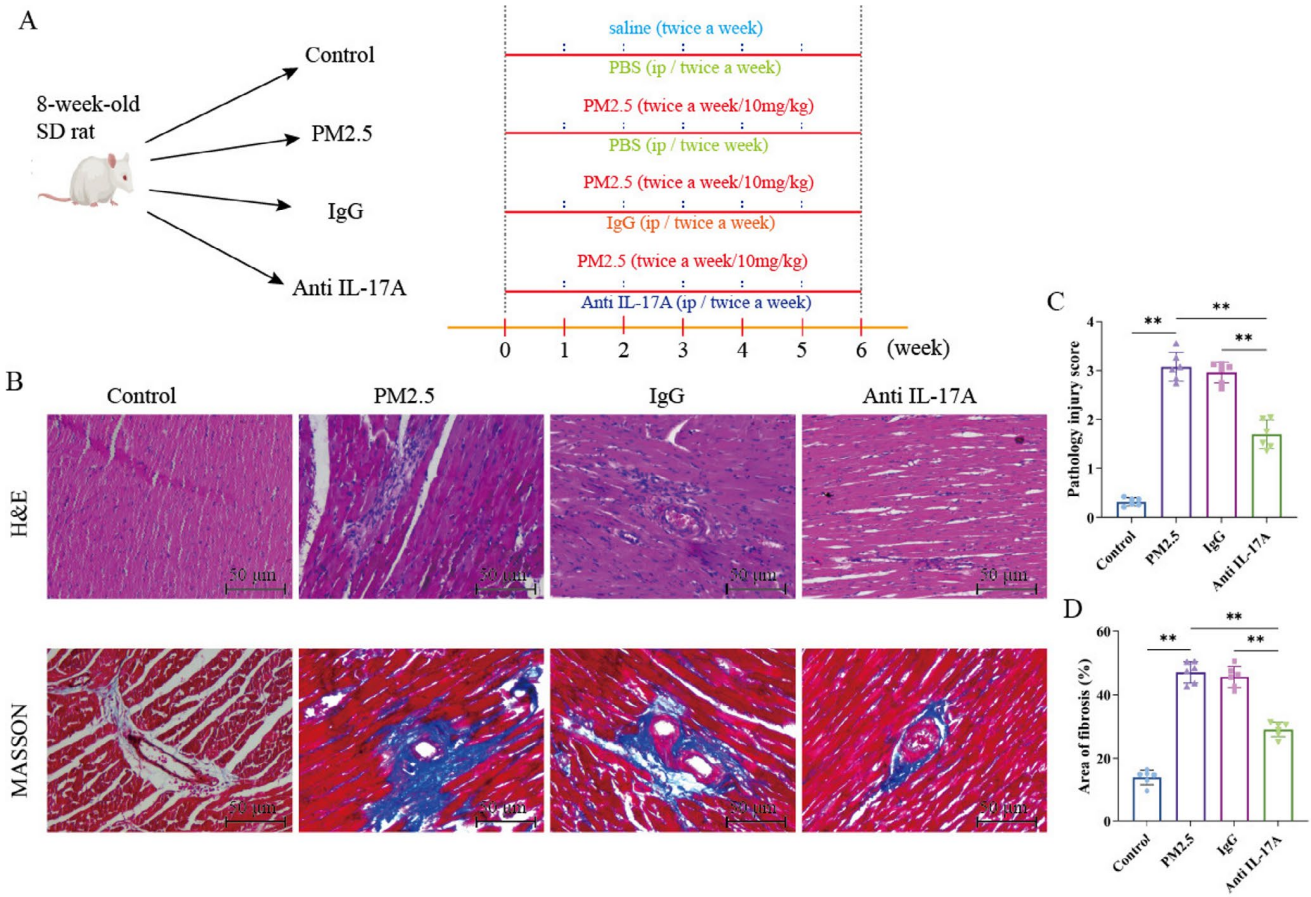
Effects of IL‐17A neutralization on cardiac pathology in PM2.5‐exposed rats. (A) Timeline for PM2.5 exposure and IL‐17A antibody treatment. (B) Representative cardiac sections stained with H&E and Masson's trichrome. (C) Cardiac pathological injury score. (D) Fibrotic area. Data are presented as the mean ± SD (*n* = 6), **p < 0.01.

### 3.3 IL‐17A Neutralization Attenuates Cardiac Fibrosis in PM2.5‐Exposed Rats

α‐smooth muscle actin (α‐SMA), Collagen I, and Collagen III are key molecules associated with cardiac fibrosis progression. These markers reflect fibroblast activation and extracellular matrix collagen deposition, respectively [23]. To examine their roles in PM2.5‐induced cardiac injury, their protein and gene expression levels were measured using IHC (Figure 3A–D), Western blot (Figure 3E–H), and RT‐qPCR (Figure 3I–K). IHC showed that α‐SMA was mainly deposited around blood vessels. Collagen I and Collagen III were predominantly expressed in the myocardial interstitium. Compared with the Control group, PM2.5 exposure significantly increased the expression of all three markers in heart tissue. These increases were markedly attenuated by the IL‐17A neutralizing antibody.

**FIGURE 3:**
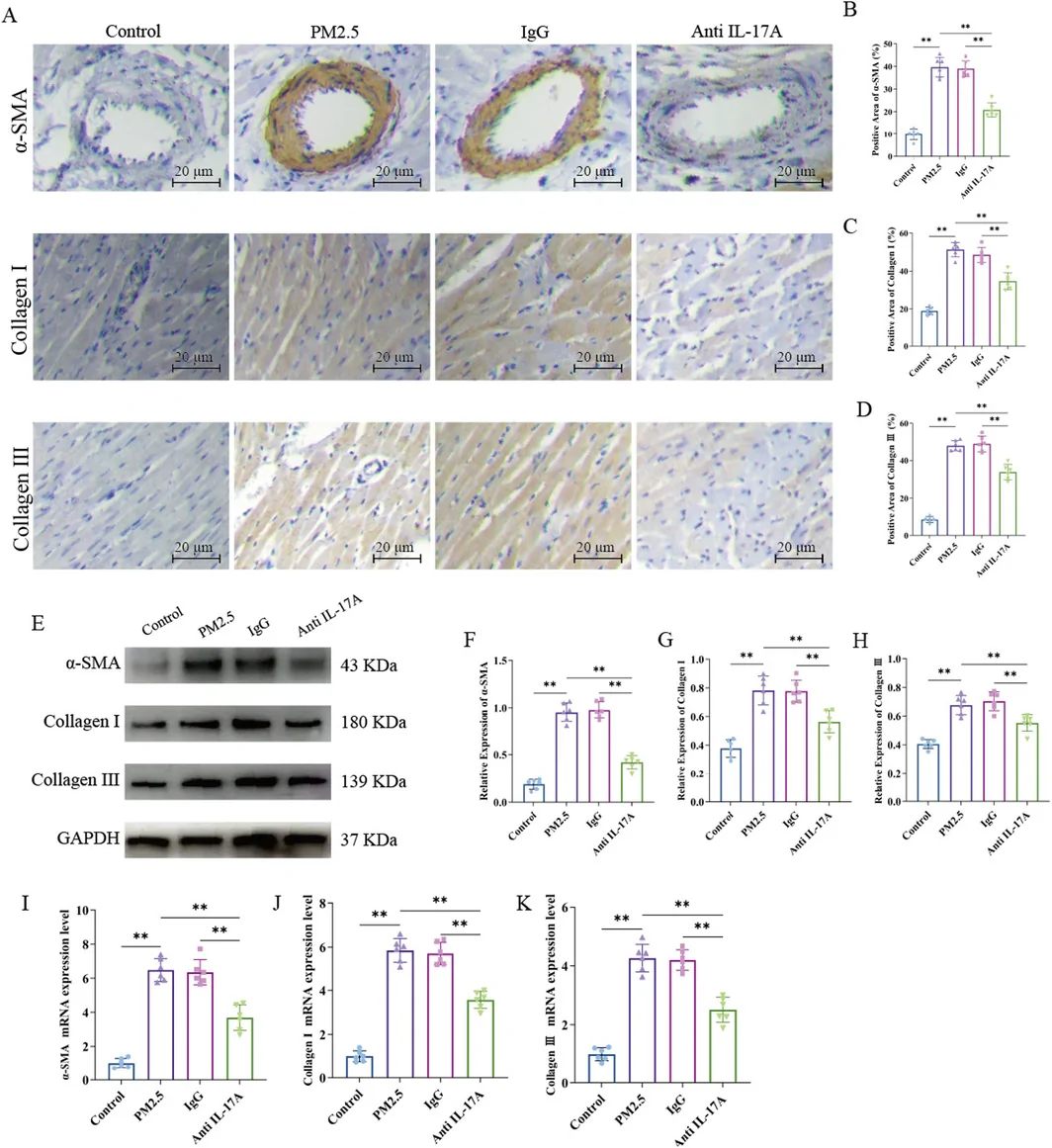
Changes in fibrosis‐related markers in cardiac tissue. (A–D) IHC images and quantification of α‐SMA, Collagen I, and Collagen III. (E–H) Western blot analysis of α‐SMA, Collagen I, and Collagen III. (I–K) Expression of mRNA encoding α‐SMA, Collagen I, and Collagen III. Data are presented as the mean ± SD (*n* = 6), **p < 0.01.

### 3.4 Neutralization of IL‐17A Improves Cardiac Function in PM2.5‐Exposed Rats

LVEF and LVFS are primary measures of cardiac systolic function. LVEDD and LVESD reflect ventricular volume load and post‐diastolic residual blood volume. Increases in these parameters suggest ventricular dilation [24]. The ratio of left ventricular mass to body weight (LV mass/BW) provides an indirect measure of cardiac hypertrophy [25]. Echocardiography (Figure 4A–F) showed that compared with the Control group, the PM2.5 group had significantly lower LVEF and LVFS. This group also had significantly higher LVEDD, LVESD, and LV mass/BW. These changes indicate that PM2.5 exposure causes cardiac dysfunction, left ventricular dilation, and myocardial hypertrophy in rats. After treatment with the IL‐17A neutralizing antibody, all of these parameters improved. In addition, serum levels of the cardiac injury markers AST and LDH were significantly higher in the PM2.5 group than in the Control group. Both markers decreased markedly following treatment with the neutralizing antibody (Figure 4G,H).

**FIGURE 4:**
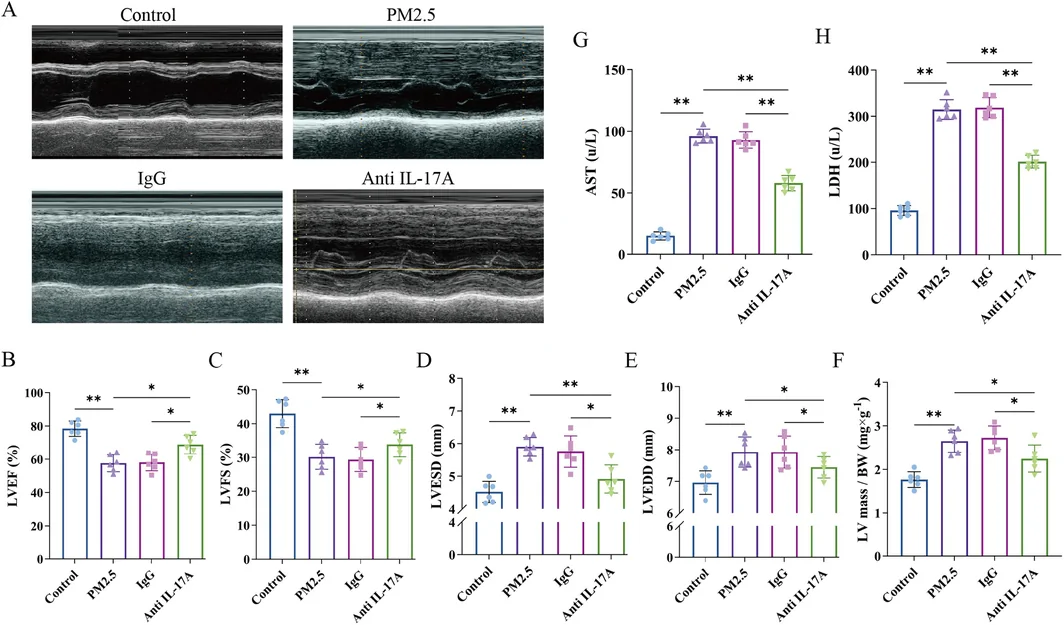
Cardiac function and injury markers in rats. (A) Representative cardiac echocardiograms. (B–F) LVEF, LVFS, LVEDD, LVESD, and LV mass/BW. (G–H) Serum concentrations of AST and LDH. Data are presented as the mean ± SD (*n* = 6), *p < 0.05, **p < 0.01.

### 3.5 IL‐17A Neutralization Alleviates Cardiac Inflammation and Oxidative Damage in PM2.5‐Exposed Rats

Inflammation and oxidative stress are key features of PM2.5‐induced cardiac injury. PM2.5 exposure disrupts the balance between ROS and the endogenous antioxidant defense system. The resulting imbalance causes oxidative stress and cellular damage [7, 26]. Additionally, inflammatory cytokines can aggravate cardiac injury by activating oxidative stress, promoting extracellular matrix remodeling, and inducing cardiomyocyte death [27]. Our data showed that the expression of p‐NF‐κB, IL‐6, and IL‐1β in cardiac tissue was significantly higher in the PM2.5 group than in the Control group. Administration of the IL‐17A neutralizing antibody markedly attenuated cardiac inflammation (Figure 5A–F). Compared with control animals, those exposed to PM2.5 showed a marked increase in ROS production. These animals also had decreased activities of SOD and GSH, elevated MDA content, and downregulated expression of SOD1 and Nrf‐2 in the heart. Overall, IL‐17A neutralization reversed these changes in oxidative stress. This reversal was reflected in restored SOD and GSH activities, decreased MDA levels, upregulated SOD1 and Nrf‐2 protein expression, and a concurrent decrease in ROS generation (Figure 5G–M).

**FIGURE 5:**
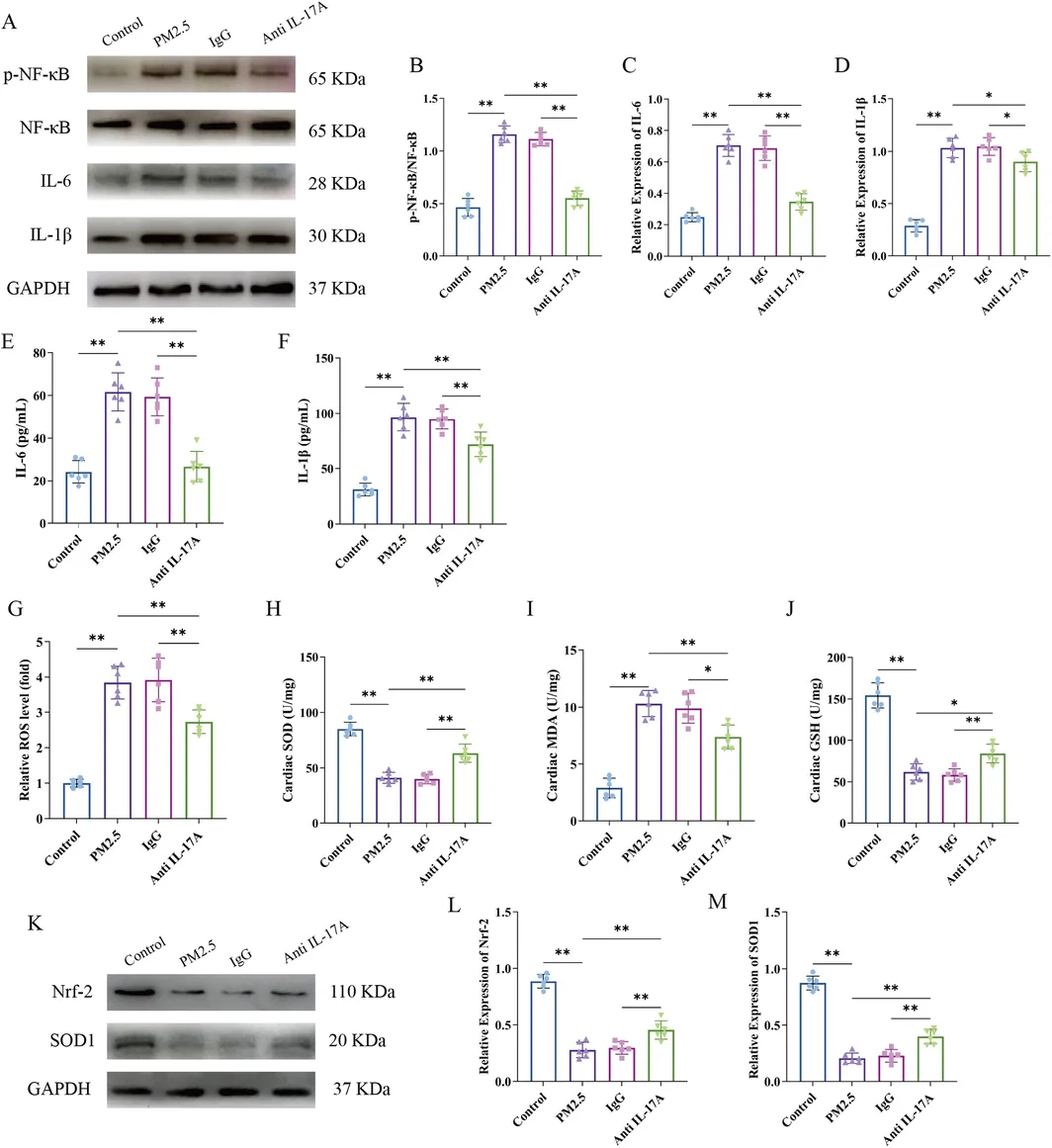
Changes in inflammation‐ and oxidative stress‐related indicators in cardiac tissue. (A–D) Western blot analysis of p‐NF‐κB, IL‐6, and IL‐1β. (E, F) ELISA measurements of IL‐6 and IL‐1β. (G–J) Activities of ROS, SOD, GSH, and MDA. (K–M) Western blot analysis of Nrf‐2 and SOD1. Data are presented as the mean ± SD (*n* = 6), *p < 0.05, **p < 0.01.

### 3.6 IL‐17A Neutralization Reduces TXNIP Expression in the Heart of PM2.5‐Exposed Rats

An exacerbated inflammatory response and increased oxidative stress can upregulate TXNIP expression [28]. To clarify how the NLRP3 inflammasome is activated, cardiac TXNIP expression was assessed using IHC (Figure 6A,B), Western blot (Figure 6C,D), and RT‐qPCR (Figure 6E). The results showed that compared with the Control group, cardiac TXNIP expression increased significantly after PM2.5 exposure. Additionally, treatment with the IL‐17A neutralizing antibody led to a significant reduction in cardiac TXNIP expression.

**FIGURE 6:**
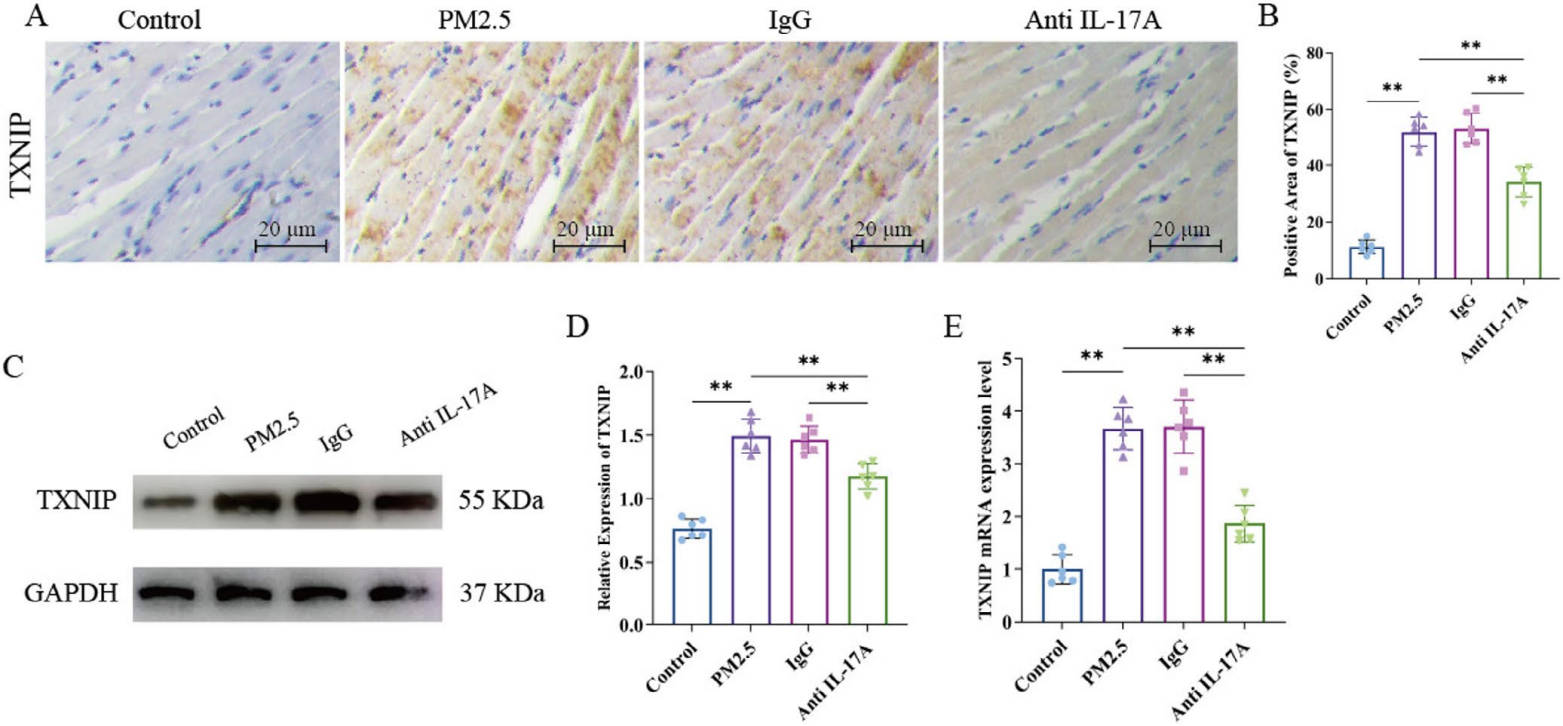
TXNIP expression in rat cardiac tissue. (A, B) IHC images and quantification. (C, D) Western blots and quantification. (E) *TXNIP* mRNA expression levels. Data are presented as the mean ± SD (*n* = 6), **p < 0.01.

### 3.7 IL‐17A Neutralization Suppresses NLRP3 Inflammasome‐Mediated Pyroptosis in the Heart of PM2.5‐Exposed Rats

Pyroptosis is a form of programmed cell death linked to inflammasome activation. In the canonical pathway, the activated NLRP3 inflammasome recruits the adaptor protein apoptosis‐associated speck‐like protein containing a CARD (ASC), which then promotes proteolytic conversion of pro‐Caspase‐1 into its active form, cleaved Caspase‐1 (p20). Active Caspase‐1 cleaves Gasdermin D (GSDMD) and releases the N‐terminal pore‐forming fragment (GSDMD‐N), which induces pyroptotic cell lysis and also promotes the maturation and secretion of the pro‐inflammatory cytokine IL‐18 [29]. To assess whether IL‐17A neutralization affects this pyroptotic cascade after PM2.5 exposure, we measured cardiac levels of NLRP3, ASC, pro‐Caspase‐1, cleaved Caspase‐1 (p20), full‐length GSDMD (GSDMD‐FL), GSDMD‐N, and mature IL‐18 by Western blot. Exposure to PM2.5 significantly increased protein levels of NLRP3, ASC, cleaved Caspase‐1, GSDMD‐N, and mature IL‐18, whereas IL‐17A antibody treatment markedly reduced their expression (Figure 7). In addition, the full‐length forms of Caspase‐1 (pro‐Caspase‐1) and GSDMD (GSDMD‐FL) increased following PM2.5 exposure and decreased following IL‐17A neutralization (Supplementary Figure 2).

**FIGURE 7:**
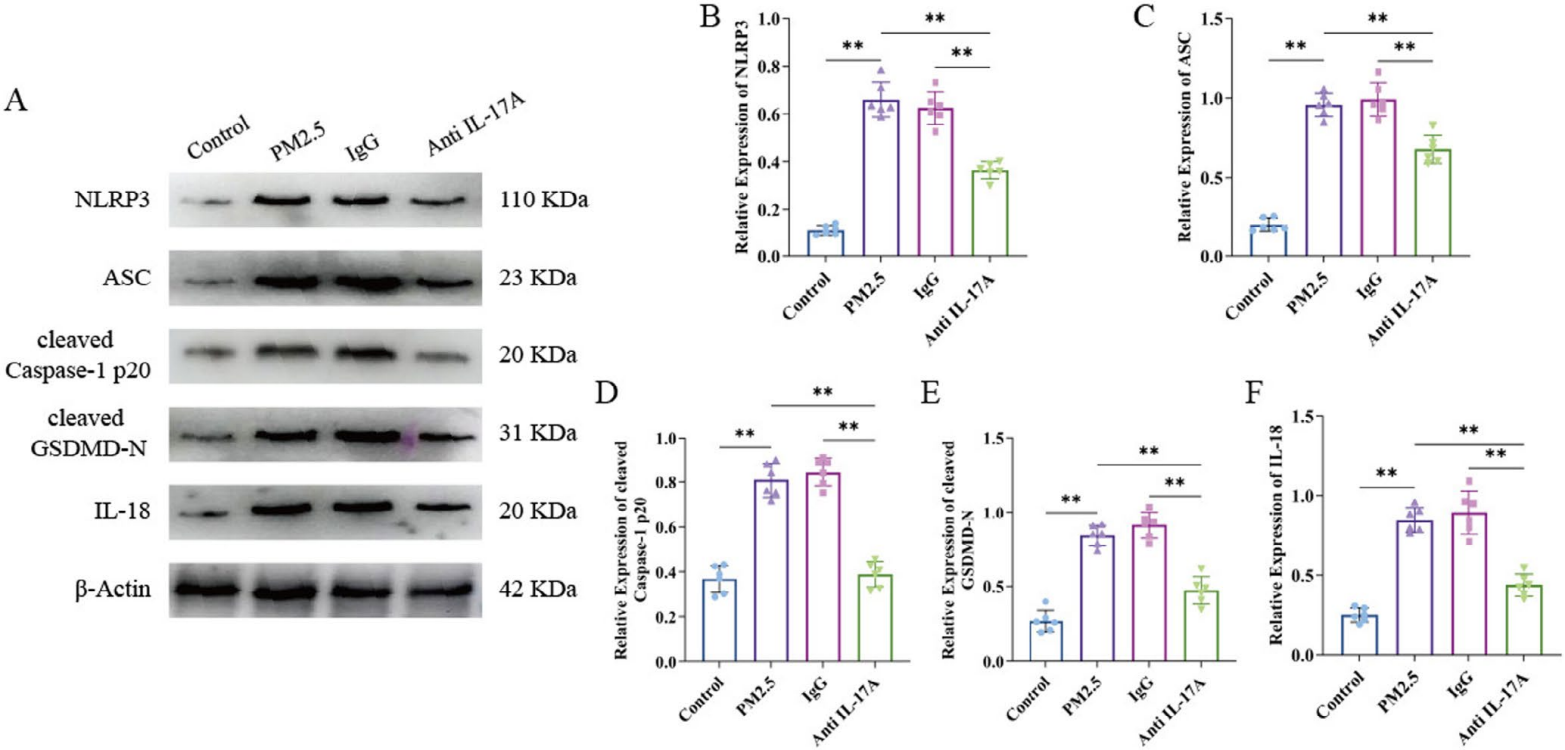
Changes in cardiac indicators related to the NLRP3 inflammasome. (A–F) Western blot analysis of NLRP3, ASC, cleaved Caspase‐1 p20, cleaved GSDMD‐N, and IL‐18 in cardiac tissue. Data are presented as the mean ± SD (*n* = 6). **p < 0.01.

### 3.8 IL‐17A Promotes Cardiac Inflammation and Fibrosis via Activation of the TXNIP/NLRP3 Pathway

To assess how IL‐17A regulates cardiac fibrosis, NRCFs were stimulated with recombinant IL‐17A in vitro. Western blot analysis (Figure 8A,B) showed that IL‐17A treatment significantly increased the expression of fibrosis‐related proteins (α‐SMA and Collagen III) and inflammatory cytokines (IL‐6 and IL‐1β). Similarly, IL‐17A stimulation markedly increased both mRNA and protein levels of TXNIP and NLRP3 (Figure 8C,D). To test the functional contribution of this pathway to the pro‐fibrotic effects of IL‐17A, NRCFs were transfected with siTXNIP to knock down TXNIP/NLRP3 pathway expression (Figure 8E,F). The transfected cells were then challenged with IL‐17A. TXNIP knockdown attenuated IL‐17A‐induced inflammatory responses and fibrotic phenotypes (Figure 8G,H).

**FIGURE 8:**
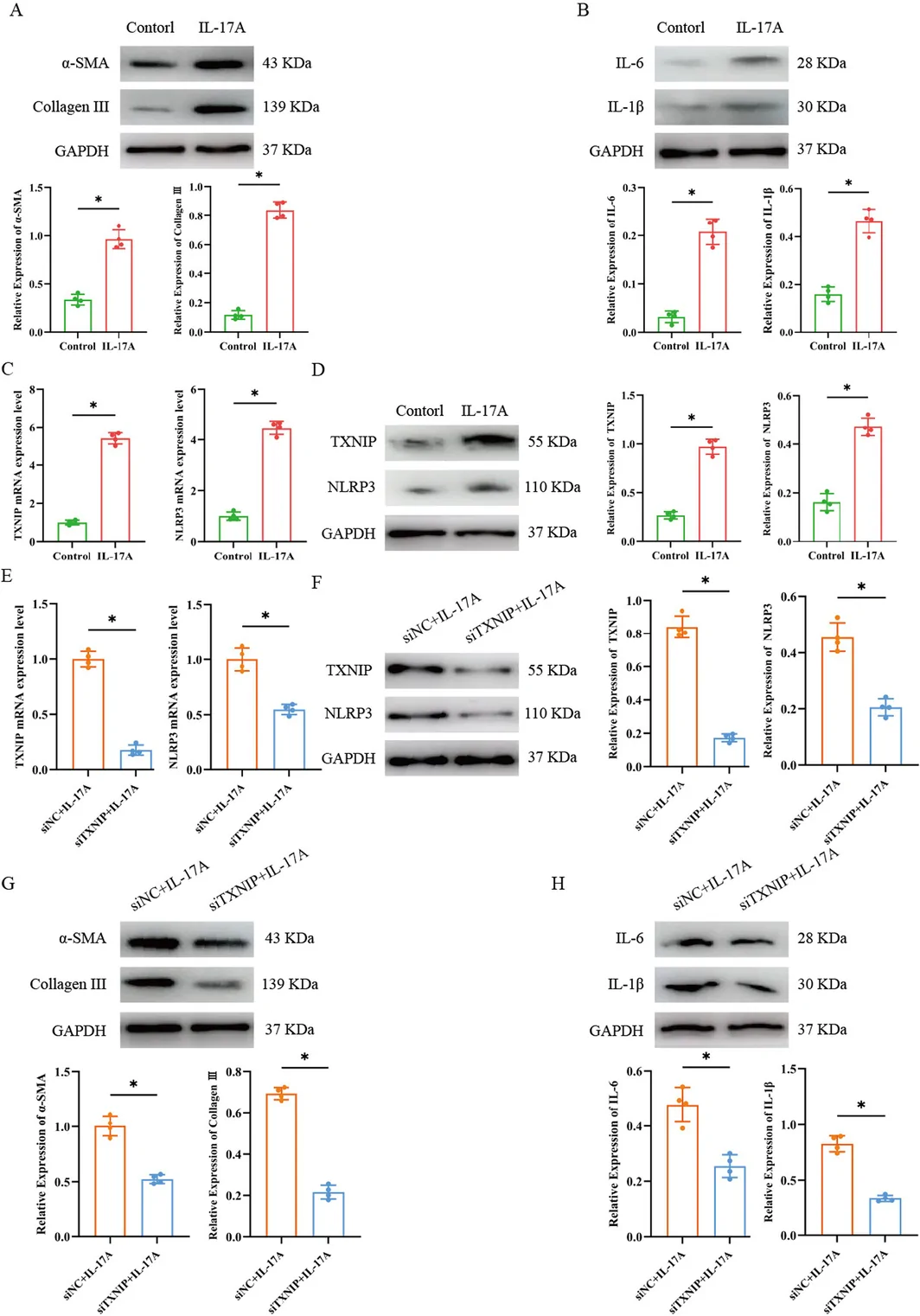
IL‐17A promotes cardiac fibrosis through the TXNIP/NLRP3 signaling pathway. NRCFs were treated with IL‐17A (100 ng/mL) for 24 h in vitro. (A, B) The protein levels of α‐SMA, Collagen III, IL‐6, and IL‐1β in the control and IL‐17A‐treated groups were measured by Western blot. (C, D) The mRNA and protein levels of TXNIP and NLRP3 in the two groups were quantified by RT‐qPCR and Western blot, respectively. In separate experiments, NRCFs were transfected with siNC or siTXNIP for 48 h, followed by treatment with IL‐17A for 24 h. (E, F) mRNA and protein expression of TXNIP and NLRP3 in the siNC and siTXNIP groups were assessed; (G, H) protein levels of α‐SMA, Collagen III, IL‐6, and IL‐1β in these two groups were measured as well. Data are presented as the mean ± SD (*n* = 4), *p < 0.05.

## 4 Discussion

PM2.5 pollution is an established environmental risk factor for cardiovascular diseases [3]. Although epidemiological evidence is strong, the molecular mechanisms underlying PM2.5‐induced cardiac inflammation and fibrosis are not fully understood, particularly how immune cell‐derived cytokines regulate this process, which remains under debate. IL‐17A is a signature pro‐inflammatory cytokine secreted by Th17 cells. However, direct evidence for the involvement of this cytokine in PM2.5‐induced cardiac injury, as well as its functional links to oxidative stress and inflammasome pathways, has been lacking. Previous studies have shown that PM2.5 exposure can activate local Th17 responses in the lung and increase IL‐17A levels in the circulation and cardiac tissue [30]. Similarly, we observed a significantly increased proportion of splenic CD4^+^IL‐17A^+^ T cells and upregulation of IL‐17A and its downstream signaling molecule TRAF6 in cardiac tissue following intratracheal instillation of PM2.5. TRAF6 is a key adaptor protein downstream of the IL‐17A receptor, and its K63 ubiquitination directly activates NF‐κB, initiating the transcription of inflammatory genes. In the heart, excessive TRAF6 activation has been implicated in cardiac hypertrophy and fibrosis [31]. Therefore, PM2.5‐induced activation of the IL‐17A/TRAF6 axis may act upstream to trigger the local cardiac inflammatory cascade. Here, the IL‐17A neutralizing antibody reduced the proportion of splenic Th17 cells and significantly downregulated cardiac TRAF6 expression, suggesting that the antibody may attenuate the direct or indirect effects of IL‐17A on the heart by suppressing the systemic IL‐17A response.

Cardiac fibrosis is a common pathological feature underlying the progression of cardiovascular diseases to heart failure. It is characterized by the activation of cardiac fibroblasts into myofibroblasts that highly express α‐SMA and the excessive deposition of extracellular matrix, composed mainly of Collagen I and Collagen III [32]. In this study, rats exposed to PM2.5 exhibited marked cardiac fibrosis, with significantly increased protein expression of Collagen I and Collagen III, consistent with previous findings [7, 33]. Reports suggest that IL‐17A may contribute to fibrosis after myocardial infarction by promoting fibroblast proliferation and upregulating transforming growth factor‐beta 1 (TGF‐β1) expression [34]. Our previous study found that blocking the IL‐17A pathway downregulated the expression of fibrosis‐related factors such as α‐SMA and Collagen III by inhibiting the TGF‐β1/Smad2/3 signaling pathway [10]. Additionally, IL‐17A neutralization attenuated PM2.5‐induced cardiac oxidative stress and histopathological injury. The in vivo experiments reported here extend these findings, demonstrating for the first time the anti‐fibrotic effects of blocking IL‐17A signaling in a PM2.5 exposure model.

Previous studies have implicated aberrant TXNIP/NLRP3 inflammasome activation in the progression of fibrosis in multiple organs and identified this pathway as a link between metabolic disorders, redox imbalance, and inflammatory injury [35, 36]. After identifying IL‐17A as a key mediator of PM2.5‐induced cardiac inflammation and fibrotic injury, we examined the mechanisms underlying its downstream effects. In vivo intervention experiments demonstrated that neutralizing IL‐17A suppressed aberrant TXNIP activation triggered by PM2.5 and subsequent NLRP3 inflammasome activation and cardiac pyroptosis. In vitro gene silencing provided functional evidence that TXNIP knockdown attenuated the pro‐inflammatory and pro‐fibrotic effects of IL‐17A on cardiac fibroblasts. These findings extend the current understanding of molecular mechanisms underlying PM2.5‐induced cardiotoxicity. Li et al. showed that PM2.5 induces myocardial fibrosis via the CHOP/TXNIP/NLRP3 pathway; however, the upstream immune regulatory signal of this pathway has not yet been elucidated [7]. The present study confirms that IL‐17A is a key upstream immunoregulatory molecule of the TXNIP/NLRP3 pathway and elucidates the crosstalk among immune inflammation, oxidative stress, and inflammasome activation in PM2.5‐induced cardiac injury. Similarly, Sivakumar et al. found that PM2.5 induces cardiac redox imbalance and myocardial injury by regulating the PI3K/AKT/mTOR/NF‐κB pathway [37]. These findings complement our results and broaden our understanding of the molecular regulation of PM2.5‐induced cardiac injury, providing a new immunological explanation for air pollution‐associated myocardial injury.

Echocardiography showed that after 6 weeks of PM2.5 exposure, rats developed left ventricular systolic dysfunction (decreased LVEF and LVFS) and eccentric remodeling (increased LVEDD, LVESD, and LV mass/BW), consistent with clinical observations of cardiac diastolic and systolic dysfunction induced by chronic PM2.5 exposure [7, 38]. After treatment with the IL‐17A neutralizing antibody, these functional parameters improved significantly, and the serum levels of cardiac injury markers AST and LDH were reduced. This suggests that despite continued PM2.5 exposure, blocking IL‐17A signaling alone can partially reverse cardiac functional impairment. In terms of public health, populations living in air‐polluted areas may experience chronic exposure to low levels of PM2.5; whether IL‐17A antibody intervention is appropriate for these populations requires further validation in long‐term animal studies. Moreover, monoclonal antibodies targeting IL‐17A (e.g., secukinumab) are available clinically to treat autoimmune diseases such as psoriasis, and preliminary data on their cardiovascular safety have been reported [39]. The present study provides a theoretical basis for repurposing these drugs to treat PM2.5 exposure‐related cardiac injury. However, systemic IL‐17A blockade may increase the risk of infection, and local cardiac delivery or the development of small‐molecule inhibitors may offer safer alternatives.

This study has several limitations. First, the IL‐17A–TXNIP/NLRP3 link was inferred primarily from parallel changes in protein/mRNA levels; direct biochemical evidence (e.g., Co‐IP for TXNIP–NLRP3 binding or Trx–TXNIP dissociation) and gain‐of‐function rescue experiments were not performed. Thus, further work is needed to validate the direct interaction and causal relationship. Second, antibody neutralization may have off‐target effects and cannot identify the specific cellular source of IL‐17A. Third, intervention and observation lasted only 6 weeks; whether long‐term IL‐17A signaling blockade leads to immunodeficiency or increased infection risk needs further assessment. Fourth, we did not distinguish the effects of IL‐17A on specific cardiac cell types, including effects on cardiomyocytes, infiltrating immune cells, and endothelial cells. Therefore, the relative contributions of different cell types remain incompletely defined. Future studies using cell‐specific gene knockout, co‐culture systems, and single‐cell sequencing are needed to clarify the role of IL‐17A in distinct cardiac cell types. Finally, the findings of this preclinical proof‐of‐concept study cannot be directly extrapolated to predict human outcomes. Clinical translation will require validation in more complex models that recapitulate disease heterogeneity and subsequently in studies of human patients.

## 5 Conclusion

In summary, IL‐17A is a major driver of PM2.5‐induced cardiac inflammation and fibrosis. IL‐17A neutralization reduces PM2.5‐induced cardiac inflammation and fibrosis in rats by suppressing the TXNIP/NLRP3 signaling pathway. These results suggest that targeting the IL‐17A–TXNIP/NLRP3 axis may offer a new treatment approach for PM2.5‐related cardiac injury.

## Funding

This work was supported by Medical Special Cultivation Project of Anhui University of Science and Technology (Grant No. YZ2023H1A007) and The Open Fund Project of Anhui Provincial Key Laboratory of Industrial Dust Deep Purification and Occupational Health Safety (Grant No. AYZJSGXLK202202005).

## Ethics Statement

This article includes no studies involving human participants conducted by any of the authors. All procedures complied with the ARRIVE guidelines and the UK Animals (Scientific Procedures) Act 1986, following approval by the Biomedical Research Ethics Committee of Anhui University of Science and Technology (Approval No.: SZ2023‐027).

## Conflicts of Interest

The authors declare no conflicts of interest.

## Supporting information


**Supplementary Figure 1.** Characterization of PM2.5. (A) Major chemical components of PM2.5, including OC, EC, water‐soluble ions, and heavy metals (HM). (B) Relative contributions of individual HM elements in PM2.5. (C) Hydrodynamic size distribution of PM2.5 suspension in 0.9% normal saline, measured by dynamic light scattering (DLS).
**Supplementary Figure 2**. Changes in pro‐Caspase‐1 and GSDMD‐FL protein levels in the heart. (A) Representative Western blots of pro‐Caspase‐1 and GSDMD‐FL in cardiac tissue. (B, C) Western blot analysis of pro‐Caspase‐1 p20 and GSDMD‐FL. Data are expressed as mean ± SD (*n* = 6), **p < 0.01.

## Data Availability

The data that support the findings of this study are available from the corresponding author upon reasonable request.
