# Supplementary material for: Neutralization of IL‐17A Ameliorates PM2.5‐Induced Cardiac Inflammation and Fibrosis in Rats by Suppressing the TXNIP/NLRP3 Signaling Pathway
Source: Kaohsiung J Med Sci. 2026 Nov 6:e70316. Online ahead of print. doi: 10.1002/kjm2.70316 (PMC13742597; doi:10.1002/kjm2.70316)
Supplement: Supplementary file 1 [file KJM2-9999-e70316-s001.docx]

**Supplementary Materials**

The chemical composition of PM2.5 was characterized as follows: elemental composition was determined by inductively coupled plasma mass spectrometry (ICP-MS; SUPEC 7000, China); water-soluble ions were measured by ion chromatography (CIC-D260, Qingdao Shenghan, China); and organic carbon (OC) and elemental carbon (EC) were analyzed using a thermal-optical carbon analyzer (OCEC-100, China) according to the NIOSH protocol. In addition, the hydrodynamic size distribution of PM2.5 particles suspended in 0.9% normal saline was determined by dynamic light scattering (DLS; BeNano 180 Zeta Max, China) at 25 °C.

**Supplementary Figure 1**

**Supplementary Figure 1** Characterization of PM2.5. (A) Major chemical components of PM2.5, including OC, EC, water-soluble ions, and heavy metals (HM). (B) Relative contributions of individual HM elements in PM2.5. (C) Hydrodynamic size distribution of PM2.5 suspension in 0.9% normal saline, measured by dynamic light scattering (DLS).

**Supplementary Figure 2**

**Supplementary Figure 2** Changes in pro-Caspase-1 and GSDMD-FL protein levels in the heart. (A) Representative Western blots of pro-Caspase-1 and GSDMD-FL in cardiac tissue. (B-C) Western blot analysis of pro-Caspase-1 p20 and GSDMD-FL. Data are expressed as mean ± SD (n = 6). **P < 0.01.
